# Correction to: Comparative Genomics and Phylogenomics of the Mustelinae Lineage (Mustelidae, Carnivora)

**DOI:** 10.1093/gbe/evag188

**Published:** 2026-07-25

This is a correction to: Azamat A Totikov, Andrey A Tomarovsky, Polina L Perelman, Tatiana M Bulyonkova, Natalia A Serdyukova, Aliya R Yakupova, David Mohr, Daniel W Foerster, Jose Horacio Grau Jipoulou, Violetta R Beklemisheva, Mikhail Sidorov, Inês Miranda, Liliana Farelo, Alexei V Abramov, Ksenia Krasheninnikova, Anna S Mukhacheva, Victor V Panov, Elena Balanovska, Nikolay Cherkasov, Karol Zub, Alan F Scott, José Melo-Ferreira, Innokentiy M Okhlopkov, Anna Zhuk, Klaus-Peter Koepfli, Alexander S Graphodatsky, Sergei Kliver, Comparative Genomics and Phylogenomics of the Mustelinae Lineage (Mustelidae, Carnivora), *Genome Biology and Evolution*, Volume 18, Issue 3, March 2026, evag014, https://doi.org/10.1093/gbe/evag014

In the originally published version of this manuscript, the affiliation provided for the fourth co-author was incorrect.

This affiliation has been corrected to read “Laboratory of System Dynamics, A. P. Ershov Institute of Informatics Systems, 630090 Novosibirsk, Pr. Lavrentieva, 6, Russia” instead of “Youth Laboratory of Molecular Genetics, Yugra State University, Khanty-Mansiysk 628011, Russia”.

